# Multidrug-Resistant *Acinetobacter* Extremity Infections in Soldiers

**DOI:** 10.3201/1108.050103

**Published:** 2005-08

**Authors:** Kepler A. Davis, Kimberly A. Moran, C. Kenneth McAllister, Paula J. Gray

**Affiliations:** * Brooke Army Medical Center, Fort Sam Houston, Texas, USA;; † Walter Reed Army Medical Center, Washington, DC, USA

**Keywords:** Acinetobacter, osteomyelitis, multi-drug resistance, wound infection

## Abstract

War wound infection and osteomyelitis caused by multidrug-resistant (MDR) *Acinetobacter* species have been prevalent during the 2003–2005 military operations in Iraq. Twenty-three soldiers wounded in Iraq and subsequently admitted to our facility from March 2003 to May 2004 had wound cultures positive for *Acinetobacter calcoaceticus-baumannii* complex. Eighteen had osteomyelitis, 2 burn infection, and 3 deep wound infection. Primary therapy for these infections was directed antimicrobial agents for an average of 6 weeks. All soldiers initially improved, regardless of the specific type of therapy. Patients were followed up to 23 months after completing therapy, and none had recurrent infection with *Acinetobacter* species. Despite the drug resistance that infecting organisms demonstrated in this series, a regimen of carefully selected extended antimicrobial-drug therapy appears effective for osteomyelitis caused by MDR *Acinetobacter* spp.

## Abstract 2

*Acinetobacter* osteomyelitis appears suppressed with extended antimicrobial drug therapy based on susceptibility patterns.

Casualty statistics from the 2003–2005 military operations in Iraq show an increase in the ratio of wounded to fatal casualties compared to previous operations in the Persian Gulf, Vietnam, and Korea (1). This relative increase of wounded casualties has led to an increased incidence of war wound infection and osteomyelitis, especially caused by multidrug-resistant (MDR) *Acinetobacter* species. The incidence of bacteremia at military medical facilities caused by *Acinetobacter baumannii* has also increased (2). The current incidence of infection with *Acinetobacter* should not be surprising. These organisms were the most frequently recovered gram-negative isolate from war wounds and the second most frequent bacterium causing bloodstream infection in US Marines with extremity wounds during the Vietnam War (3). In nonconflict environments, *Acinetobacter* species are rarely responsible for community-acquired infections. In the hospital setting, *Acinetobacter* species are an important cause of nosocomial infection, yet these infections were rarely encountered in our facility until we began observing them in soldiers with infected wounds. Nosocomial infections caused by *Acinetobacter* species include pneumonia, meningitis, bloodstream, urinary tract, surgical wound, and soft tissue infections (4). Such infections are challenging to treat because of extensive antimicrobial drug resistance. Osteomyelitis caused by *Acinetobacter* occurs, but it is less frequently reported and had not been identified in our facility during the 14 months before March 2003. Optimal therapy for osteomyelitis caused by these organisms is not well defined because of limited available data. This case series reviews 1 military medical center's experience with these infections, including species identified, antimicrobial drug–susceptibility patterns, antimicrobial drug therapy, and clinical outcomes.

## Methods

Case reports were compiled from active-duty soldiers admitted to Brooke Army Medical Center (BAMC) in San Antonio, Texas. This tertiary military medical center serves a population of active-duty and retired soldiers and their dependents along with a limited number of civilian trauma patients admitted from the local area. The hospital was operating at an average capacity of 175 beds during the study period. This facility also houses the US Army's Institute of Surgical Research, which treats both active-duty and civilian trauma patients with burn injuries. Data collection for this case series was completed under a study protocol approved by BAMC's Department of Clinical Investigation Institutional Review Board.

### Identification of Patients

All wound, sputum, urine, and blood culture results completed at our hospital from March 1, 2003, to May 31, 2004, were reviewed. Those patients who had *Acinetobacter*-positive cultures were then compared to all active-duty soldiers admitted to our facility. A soldier was considered for inclusion if he had an *Acinetobacter*-positive culture and had been deployed to Iraq or Afghanistan and had an admission diagnosis of injury (ICD codes 800.0–900.0). Similarly, hospital admission and laboratory data were reviewed for the 14 months before the study period to define the incidence of *Acinetobacter* infection in hospitalized, active-duty soldiers before the onset of military action in Iraq.

### Case Definitions

Patients with either *Acinetobacter* contiguous focus osteomyelitis or wound infection are included in this series. Cases were defined as osteomyelitis if bone tissue collected during surgical procedures (primarily open debridements but also including placement of external or internal fixators or bone grafting) was positive for *Acinetobacter* spp. on routine culture (5,6). In addition, patients with open fractures or exposed bone with gross findings of infection (purulence, necrotic tissue, or environmental contamination with exposed bone), clinical evidence of infection (temperature >38°C, leukocyte count >12,000/μL), and *Acinetobacter* spp. identified from culture of deep wound tissue obtained intraoperatively, excluding bone, were also defined as having osteomyelitis (7). Cases were defined as wound infection if similar deep wound cultures were positive for *Acinetobacter* spp. with gross findings and clinical evidence of infection but no exposed bone and no fracture. Colonization with *Acinetobacter* was defined as a positive culture for *Acinetobacter* without gross findings or clinical evidence for infection.

The *Acinetobacter* isolate was defined as MDR if it was resistant to ≥3 classes of antimicrobial agents as tested by automated antimicrobial drug–susceptibility testing (Vitek, bioMérieux, Hazelwood, MO, USA) (8). On occasion, isolates were further evaluated with disk diffusion antimicrobial testing for susceptibilities to alternate antimicrobial drugs, such as colistin, or to confirm automated susceptibility results. Confirmatory disk diffusion susceptibility testing was completed only for those isolates that were resistant to all antimicrobial agents by automated testing or if only 1 antimicrobial drug was listed as susceptible. Disk diffusion testing was performed in accordance with Clinical and Laboratory Standards Institute (formerly NCCLS) guidelines (9). Colistin susceptibility was assumed if the zone of inhibition was ≥14 mm (10).

Patients were evaluated for recurrence of infection. Many patients underwent subsequent reconstructive surgeries, and the bone tissue was sent for culture. Definitions of recurrent infection followed the previously described criteria for the case definitions with the following additions: recurrent infection was defined as having *Acinetobacter* spp. isolated at the original site of infection after completing an antimicrobial drug treatment course for the initial infection; secondary infection was defined as infection with a different organism at the same site as the initial *Acinetobacter* infection.

### Data Collection

Both electronic and paper charts of all patients who met case definition criteria were retrospectively reviewed for demographic, diagnostic, and treatment data. Laboratory results were reviewed for *Acinetobacter* species isolated and antimicrobial drug susceptibilities. Patients were also interviewed either in person or by telephone to confirm mechanism of injury, length of antimicrobial drug treatment course, recurrence of infection, subsequent hospital admissions, and clinical outcome of the sustained injury and infection (resolved, continuing convalescence, or amputation). Follow-up was defined as the time from completing the initial antimicrobial treatment course to the date of the study interview.

## Results

### Case Inclusion Criteria

From March 1, 2003, to May 31, 2004, a total of 24,114 cultures (blood, urine, wound, sputum) were completed in our hospital. Of these, 145 (0.6%) were positive for *Acinetobacter* spp. During the same period, 237 active-duty patients were admitted to our facility with the admission diagnosis of injury (Figure). Of these admitted soldiers, 151 (64%) had been deployed to OIF/OEF. Cultures of blood, wound, sputum, urine, or skin were obtained for 84 of these patients; 48 (32% of admitted deployed soldiers) were positive for *Acinetobacter* spp. Of these, 30 (63%) represented clinical infection; the remaining 18 represented colonization with *Acinetobacter.* Of those patients with cultures that represented clinical infection, 23 met the case definition for *Acinetobacter* osteomyelitis (Table 1) or *Acinetobacter* wound infection (Table 2). During the 14 months before the study period, only 2 active-duty soldiers, of 326 admitted to our facility, had any *Acinetobacter* infection. The incidence of *Acinetobacter* infection during the study period represents a significant increase when compared to the control period (p<0.01 by 2-tailed Fisher exact test).

**Figure:**
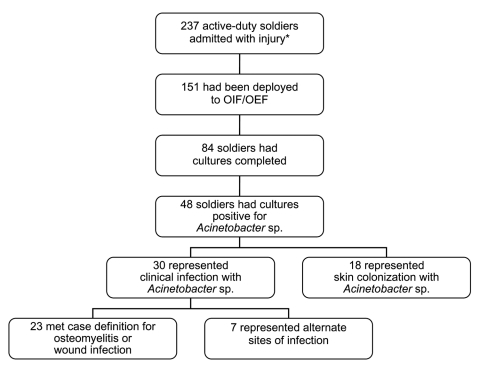
Flow chart illustrating active-duty soldier admissions to Brooke Army Medical Center from March 1, 2003, to May 31, 2004, and those who met case definitions for *Acinetobacter* osteomyelitis or wound infection. *Soldiers with diagnosis of injury, ICD codes 800.0–900.0. OIF/OEF, Operation Iraqi Freedom/Operation Enduring Freedom.

**Table 1:** Acinetobacter osteomyelitis*

Patient	Osteomyelitis location	Mechanism of injury	MDR isolate	Bacteremia	Hardware	Parenteral drug therapy	Follow on oral antimicrobial agents	Recurrent infection	Follow-up, wk†
1	Left radius/ulna	Landmine explosion, driver of pavement grater	Yes	No	Yes	Imipenem 500 mg every 6 h, amikacin 20 mg/kg/d for 8 wk	No	Secondary infection with MSSA	12
2	Right humerus/shoulder	IED round through shoulder	Yes	No	Yes	Imipenem 500 mg every 6 h, amikacin 15 mg/kg/d for 6 wk	Yes, ciprofloxacin 500 mg 2 × a day	No	32
3	Right humerus/shoulder	IED blast, shrapnel injury	Yes	No	Yes	Imipenem 500 mg every 6 h, amikacin 15 mg/kg/d for 7 wk	No	No	35
4	Left radius/ulna	Gunner in APC who received RPG blast	Yes	Yes	No	Imipenem 500 mg every 6 h for 7 wk, with amikacin 20 mg/kg/d for 3 wk changed to amp/sulb 3 g every 6 h for 4 wk	No	No	4
5	Left tibia	Mortar blast, shrapnel injuries	Yes	No	No	Ceftazidime 2 g every 8 h; amikacin 12.5 mg/kg/d for 7 wk	No	No	7
6	Right distal humerus/elbow	Passenger in HMMWV, roadside IED blast	Yes	No	Yes	Imipenem 500 mg every 6 h for 7 wk	No	Secondary infection with *Enterobacter cloacae*	22
7	Left tibia/fibula	Proximate IED blast	Yes	No	Yes	Amp/sulb 12 g continuous 24-h infusion for 6 wk	No	No	36
8	Left humerus	Proximate mortar round blast	Yes	Yes	No	Meropenem 1 g every 8 h for 7 wk	No	No	5
9	Left tibia/fibula	Driver of Humvee, hit land mine	Yes	No	No	Gentamicin 5 mg/kg/d for 3 wk	No	No	40
10	Left distal humerus/elbow	Truck driver, IED blast	Yes	No	Yes	Imipenem 500 mg every 6 h for 6 wk	No	Yes—secondary pin tract infection, No culture	30
11	Right humerus/shoulder	Gunner in HMMWV, IED blast	Yes	No	No	None	No	No	39
12	Right humerus/elbow	HMMWV passenger, IED blast	No	No	Yes	Imipenem 500 mg every 6 h, amikacin 15 mg/kg/d for 6 wk	No	Secondary infection with MRSA	48
13	Left tibia	50-caliber gunshot wound	No	No	No	Amp/sulb 3 g every 6 h, amikacin 20 mg/kg/d for 6 wk	Yes—amox/clav 875/125 mg 2 ×/d for 3 wk	Secondary infection with MSSA	61
14	Left humerus/elbow	Motor vehicle accident, elbow out window, no blast injury	No	No	Yes	Imipenem 500 mg every 6 h, gentamicin 5 mg/kg/d for 4 d	Yes—levofloxacin 500 mg/d for 10 d	No	57
15	Right femur	Passenger HMMWV, RPG round to right leg	No	Yes	No	Imipenem 500 mg every 6 h, amikacin 20 mg/kg/d for 6 wk	No	No	9
16	Left tibia	Gunshot wound to left leg	No	No	No	Imipenem 500 mg every 6 h, amikacin 15 mg/kg/d for 8 wk	No	No	35
17	Right tibia/fibula	Passenger HMMWV, IED blast, open fracture	No	No	No	Imipenem 500 mg every 6 h for 4 wk, followed by meropenem 1 g every 8 h for 2 more wk	No	Secondary infection with MRSA	50
18	Left tibia	Motor vehicle accident, run over by tank	No	No	No	Ceftazidime 2 g every 8 h for 4 wk	No	No	56

*MDR, multidrug resistant; MSSA, methicillin-sensitive *Staphylococcus aureus*; IED, improvised explosive device; APC, armored personnel carrier; RPG, rocket-propelled grenade; HMMWV, high mobility multipurpose wheeled vehicle, also known as Humvee; MRSA, methicillin-resistant *S. aureus*; Amp/sulb, ampicillin/sulbactam; Amox/clav, Amoxicillin + clavulanic acid. †Length of follow-up after completion of antimicrobial drug therapy.

**Table 2:** Acinetobacter wound infection*

Patient	Wound infection location	Mechanism of injury	MDR isolate	Bacteremia	Parenteral drug therapy	Follow on oral antimicrobial agents	Recurrent infection	Follow-up, wk†
19	Right achilles tendon wound	RPG blast wound to right Achilles in driver of HMMWV	Yes	No	Imipenem 500 mg every 6 h for 5 wk	No	Secondary infection, infected hematoma with CNS	36
20	Left thigh wound	Proximate car-bomb blast	Yes	No	Imipenem 500 mg every 6 h for 2 wk	No	No	11
21	Right elbow wound	RPG fire, with traumatic right arm amputation below elbow	Yes	No	Cefazolin 1 g every 8 h for 10 d	No	No	92
22	Scalp wound	35% TBSA burn injury, passenger in HMMWV that hit land mine	Yes	No	Imipenem 1 g every 8 h for 16 d	No	No	89
23	Hand wound	27% TBSA burn injury, passenger in HMMWV hit by RPG	Yes	No	Imipenem 500 mg every 6 h for 14 d	No	No	30

*MDR, multidrug-resistant; RPG, rocket-propelled grenade; CNS, coagulase-negative *Staphylococcus*; TBSA, total body surface area; HMMWV, high mobility multipurpose wheeled vehicle, also known as Humvee. †Length of follow up after completion of antimicrobial drug therapy.

### Demographics

All patients included in this series had been transferred to BAMC through the military airmobile medical evacuation system. All, excluding one, were evacuated through, and admitted for at least 1 day to, Landstuhl Army Medical Center in Landstuhl, Germany; 3 patients were admitted to a second US Army medical center before admission to BAMC. The median time from injury to admission at BAMC was 6 days (range 2–36 days, Table 3). The median time from injury to identification of infection was also 6 days (range 3–12 days). *Acinetobacter* infection was initially identified at BAMC in 15 of the 23 patients; the remainder were identified at a previous medical center. None were initially diagnosed prior to evacuation from Iraq or Afghanistan. The median age of the patients was 26 years (range 20–48), and all but 2 were men. Patients were generally stable on admission to BAMC and did not require admission to an intensive care unit.

**Table 3:** Patient demographics*

Patient	Age, y	Time (d) from injury to			
		BAMC admission	Diagnosis of infection	No. MC admissions before BAMC admission	Infection initially diagnosed at BAMC
1	20	13	4	1	N
2	26	10	10	1	Y
3	31	11	12	1	Y
4	21	9	7	2	N
5	21	4	5	1	Y
6	37	13	NA	2	N
7	33	5	6	1	Y
8	48	5	5	1	Y
9	21	4	NA	1	N
10	34	13	NA	1	N
11	21	6	7	1	Y
12	37	6	7	1	Y
13	22	3	3	0	Y
14	23	5	9	1	Y
15	33	3	4	1	Y
16	22	5	6	1	Y
17	27	13	NA	1	N
18	26	36	6	1	N
19	26	16	9	2	N
20	21	6	6	1	Y
21	26	5	5	1	Y
22	20	4	9	1	Y
23	24	2	10	1	Y

*BAMC, Brooke Army Medical Center; MC, medical center; N, no; Y, yes; NA, not available.

### Microbiologic Data

Patients with *Acinetobacter* osteomyelitis primarily had bone tissue collected during surgical procedures that was culture-positive for *A. calcoaceticus-baumannii* complex. This was the only species and organism identified in all initial tissue cultures. Ten patients had deep wound cultures, excluding bone tissue, that were positive for *A. calcoaceticus-baumannii* complex. Five (patient numbers 4, 9, 10, 11, and 15, Table 1) had open fractures with environmental contamination and signs of infection that met the case definition of osteomyelitis. The remaining 5 (Table 2) did not meet criteria for diagnosis of osteomyelitis and were diagnosed with wound infection. Two of these patients had burn injuries. Cultures of debrided soft tissue in these 2 patients were positive for *Acinetobacter* within the first 8 days of hospitalization, and pathologic evaluation of tissue demonstrated invasive infection. Patient no. 22 had a soft tissue wound culture positive on hospital day 5 (postinjury day 9); patient no. 23 had a soft tissue wound culture positive on hospital day 8 (postinjury day 10).

### Antimicrobial Drug–susceptibility Data

Thirty-eight cultures from the 23 patients reported in this study were positive for *Acinetobacter* spp. (Table 4). Twenty-nine isolates were MDR, as tested by automated susceptibility testing. All but 4 of the MDR isolates were susceptible to imipenem, and no imipenem resistance developed in the 15 patients who received this drug during therapy. Three of these 4 isolates were susceptible only to amikacin. Of the 25 imipenem-susceptible MDR *Acinetobacter* isolates, 10 demonstrated resistance to all other tested antimicrobial agents. Other isolates were susceptible to only 1 other antimicrobial agent: 7 were also susceptible to amikacin, 3 to ampicillin/sulbactam, 2 to tobramycin, and 1 to trimethoprim/sulfamethoxazole. Nine isolates were not MDR. These isolates were susceptible to ≥3 classes of the tested antimicrobial agents. Three MDR isolates were tested for susceptibility to colistin; all 3 were susceptible by disk diffusion testing. One was susceptible only to imipenem, 1 to amikacin alone, and 1 to both amikacin and ceftazidime.

**Table 4:** Acinetobacter calcoaceticus-baumannii complex antimicrobial drug susceptibilities for 38 isolates recovered from wound or blood cultures

Antimicrobial drug	Susceptible (%)
Amikacin	48
Amoxicillin/clavulanate	9
Ampicillin/sulbactam	50
Cefepime	14
Cefotetan	3
Ceftazidime	12
Ceftriaxone	6
Ciprofloxacin	11
Colistin*	100
Gentamicin	8
Imipenem	89
Tobramycin	14
Trimethoprim/sulfamethoxazole	29

*Colistin susceptibility evaluated in 3 multidrug-resistant isolates.

### Therapy

Antimicrobial drug treatment of these infections was based on susceptibility testing, and all patients with osteomyelitis underwent multiple surgical debridements of necrotic bone. Ten of the patients with osteomyelitis were treated with dual antimicrobial agents, 7 with monotherapy, and 1 with surgical debridement alone. Only patients with osteomyelitis received dual antimicrobial drug therapy. Of the 10 treated with dual therapy, 5 had MDR *Acinetobacter* spp. and 5 had non-MDR *Acinetobacter* spp. isolated. The primary combination of antimicrobial agents was imipenem (500 mg every 6 h) in combination with high-dose amikacin (15–20 mg/kg daily). In a few instances, when imipenem was not active against the isolated organism, ampicillin/sulbactam or ceftazidime was used if either was active against the particular isolate (Table 1). Of the 7 treated with monotherapy, 5 had MDR *Acinetobacter* isolated. All patients with wound infection received monotherapy based on antimicrobial drug–susceptibility testing results.

### Follow-up

The follow-up period was 1–23 months (mean 9 months). During this time, no *Acinetobacter* infections recurred at any site, including the bloodstream. Seven secondary infections occurred, 6 in those with an initial diagnosis of osteomyelitis and 1 with wound infection. Four occurred in patients with MDR *Acinetobacter* (3 with osteomyelitis and 1 with wound infection). These secondary infections primarily involved other resistant nosocomial pathogens (Tables 1 and 2).

### Control Period

During the 14 months before March 2003, only 2 active-duty soldiers had *Acinetobacter* infection. A soft tissue infection with *Acinetobacter* developed in 1 soldier with a history of bullous pemphigoid. Bacteremia with *Acinetobacter* developed in the other soldier, who had a history of Ewing sarcoma. The latter *Acinetobacter* isolate was not a MDR organism and was treated with imipenem (500 mg parenterally) for 14 days.

## Discussion

The 23 cases observed during the study period represent a significant increase in the incidence of clinical infection with *Acinetobacter* in our facility. Similarly, the rate of blood, wound, or urine cultures positive for *Acinetobacter* species increased 3-fold during the study period as compared to the control time period (data not shown). This increase and the influx of severe extremity infection due to MDR *Acinetobacter* species posed considerable challenges. The foremost was determining appropriate therapy for osteomyelitis caused by MDR *Acinetobacter* species without institutional or historical experience to guide us. In addition, increasing prevalence of this MDR gram-negative organism in our facility mandated new infection control procedures to limit nosocomial spread. Finally, the occurrence of *Acinetobacter* wound infection was somewhat unexpected, and initially the reservoir for infection was unclear and generated much debate. Recent investigation by the military medical and research community suggests that these are nosocomial infections; however, their exact source remains unclear.

Most *Acinetobacter* infections reported in the literature reflect nosocomial *Acinetobacter*, as hospitalized patients are at increased risk because of severe illness or disability, extremes of age, and relative states of immunocompromise (4). *Acinetobacter* species can cause infection in any organ system, including bacteremia, pneumonia, endocarditis, meningitis, urinary tract infection, intraabdominal abscess, osteomyelitis, soft tissue infection, and surgical site infections (11). Data collected from a review of sentinel hospitals in the United States demonstrated that 1.5% of all nosocomial bloodstream infections were due to *Acinetobacter* species (12). Crude death rates associated with nosocomial *Acinetobacter* infection are 19%–54% (12–15). The difficulty in treating these infections is not due to any excessive virulence of the organism per se but rather to its antimicrobial drug resistance. Many nosocomial isolates are resistant to ≥3 classes of antimicrobial agents, which classifies them as MDR organisms (8,12). A common susceptibility pattern in this case series was resistance to all antimicrobial agents except imipenem and amikacin.

When these patients were first evaluated, data to guide therapeutic decisions were limited. Previous reported experience with osteomyelitis caused by *Acinetobacter* species is scant. It has been described after a hamster bite in an 8-year-old boy (16) and in a patient who previously had an artillery fragment injury that caused an open fracture of the right femur (17). Other reviews have described osteomyelitis as a sequela of infection with *Acinetobacter* species but did not report details of therapy or followup (4,11). Patients in our case series primarily received extended dual antimicrobial–drug therapy based on susceptibility patterns of the recovered organisms. Combination therapy has been shown to decrease the risk for development of more highly resistant organisms, which has been reported when single agents are used alone (18). While on this antimicrobial regimen, patients demonstrated clinical improvement with marked reduction of inflammatory markers. Many of these patients had internal stabilizing hardware placed into the infected area at the time of diagnosis of infection. This hardware remained in place at the completion of parenteral therapy. In these situations, when the causative organism was susceptible to oral antimicrobial agents, oral suppressive therapy was continued as long as the stabilizing hardware remained in place. In most cases, however, because of extended antimicrobial drug resistance, no oral agents maintained activity against the *Acinetobacter* isolate. Once those infected with MDR isolates demonstrated clinical improvement and normalization of inflammatory markers, antimicrobial drug therapy was discontinued without continuing long-term suppressive therapy (Tables 1 and 2).

During the follow-up period, no recurrent episodes of *Acinetobacter* osteomyelitis have occurred. The relative brevity of follow-up is a limitation of this study. The ultimate outcome for these patients will not be known for many years, as they have increased risk for recurrent infection throughout their lifetime. In addition, *Acinetobacter* organisms do not possess substantial inherent virulence. None of the patients in this series failed therapy, and none died because of *Acinetobacter* infection. Such is not the case in outbreaks among immunocompromised or intensive care patients, in whom *Acinetobacter* infection leads to increased mortality (12–15). The successful outcomes in this case series may be a reflection of the youth and general good health of the soldiers infected.

MDR *Acinetobacter* is an important nosocomial pathogen with multiple recent outbreaks reported (18–22). It has the capacity to survive in dry environments (23,24), which increases the risk for nosocomial transmission. The increasing prevalence of MDR *Acinetobacter* in our facility led to new infection control procedures. Currently, all injured soldiers admitted to our facility returning from OIF/OEF are placed in contact isolation. Screening cultures of the axilla, groin, and any open wound are completed to assess for colonization with MDR *Acinetobacter*, which was identified in 18 of 151 admitted soldiers during the study period (Figure). If all cultures taken on admission are negative, the soldier is then removed from contact isolation. Soldiers with wound infection or osteomyelitis caused by MDR *Acinetobacter* are kept in contact isolation for the duration of hospitalization. Implementation of these types of infection control procedures has limited nosocomial spread in previously reported outbreaks (18,20,22), which is the goal of our current policy, in addition to controlling the continuing reservoir of this organism.

As previously noted, we initially suspected that colonized soldiers themselves were the reservoir for MDR *Acinetobacter*, and that this colonization was obtained from the environment. This hypothesis was based on 2 facts. First, these organisms are ubiquitous in the environment (4,25), and inoculation of these organisms into war wounds during traumatic blast, shrapnel, or projectile injuries seemed to be plausible. Second, *Acinetobacter* spp. had previously been described as common pathogens in war wounds (3), supporting the initial hypothesis. However, these infections are apparently similar to recently reported nosocomial MDR *Acinetobacter* infections. Investigation into the cause of these infections is ongoing, but the source is unlikely to be environmental. Multiple follow-up soil samples have not yielded *Acinetobacter*, yet it has been recovered from environmental cultures within field medical facilities. The final outcome of this investigation is pending further analysis.

Data from this case series demonstrate that highly resistant *Acinetobacter* infection, including osteomyelitis, can be successfully treated with appropriate surgical debridement, directed antimicrobial drug therapy, and careful follow-up. Our patients responded to this multifaceted approach, although their final outcome will not be determined for several years. These patients continue to be followed for recurrence of MDR *Acinetobacter* infection. Clearly, guided therapy based on antimicrobial drug susceptibility leads to suppression of recurrent infection up to 23 months. Most of the patients in this series did not receive extended continuation therapy with oral antimicrobial agents; whether such therapy would provide added benefit is unclear. However, few antimicrobial drug options are currently available, with none soon to be released, to treat infections caused by resistant gram-negative organisms. Increasing prevalence of these types of infections highlights the necessity for newer antimicrobial agents with activity against these organisms.
